# Hepatitis E Virus Outbreak among Tigray War Refugees from Ethiopia, Sudan

**DOI:** 10.3201/eid2808.220397

**Published:** 2022-08

**Authors:** Ayman Ahmed, Yousif Ali, Emmanuel Edwar Siddig, Jehan Hamed, Nouh S. Mohamed, Amna Khairy, Jakob Zinsstag

**Affiliations:** University of Khartoum, Khartoum, Sudan (A. Ahmed, E.E. Siddig, J. Hamed);; Sirius Training and Research Centre, Khartoum (A. Ahmed, N.S. Mohamed);; Swiss Tropical and Public Health Institute, Allschwil, Switzerland (A. Ahmed, J. Zinsstag);; University of Basel, Basel, Switzerland (A. Ahmed, J. Zinsstag);; Sudan Federal Ministry of Health, Khartoum (Y. Ali, A. Khairy);; Erasmus MC University Medical Center, Rotterdam, the Netherlands (E.E. Siddig)

**Keywords:** hepatitis E virus, viruses, zoonoses, enteric infections, acute jaundice syndrome, HEV, Ethiopia, refugees, humanitarian crisis, Tigray War, Sudan

## Abstract

We report hepatitis E virus (HEV) outbreaks among refugees from Ethiopia in Sudan during June 2021–February 2022. We identified 1,589 cases of acute jaundice syndrome and used PCR to confirm HEV infection in 64% of cases. Implementing vaccination, water, sanitation, and hygiene programs might reduce HEV outbreak risk.

Hepatitis E is a hygiene- and sanitation-related disease caused by hepatitis E virus (HEV), a member of the Hepeviridae viral family (*1*,*2*). HEV has 4 genotypes: genotypes 1 and 2, predominantly found in humans, and genotypes 3 and 4, found in both humans and animals (*1*,*2*). Main zoonotic virus reservoirs include domestic pigs, wild boars, rodents, and sika deer (*2*). Risk factors for transmission differ depending on the genotype. However, genotype 1 is associated with maternal mortality, waterborne transmission, and outbreaks in Africa (*3*,*4*). In low- and middle-income countries, HEV is mainly transmitted through contaminated drinking water (*2*). The clinical manifestation of HEV infection is largely genotype-dependent (*2*–*4*).

HEV is a common cause of acute hepatitis and jaundice worldwide. The World Health Organization estimates that 20 million HEV infections (16.5% symptomatic) and 44,000 HEV-related fatalities occur annually (*2*). The public health threat of HEV infection is exceptionally high in Africa, and biennial outbreaks result in ≈35,300 cases of infection and 650 fatalities (*3*). Pregnant women in Africa are at higher risk for HEV infection than other persons and have an HEV-related mortality rate 10 times higher than the general population (*4*). Outbreaks of HEV infections in Africa are associated with camps for refugees and internally displaced persons (*4*). Limited knowledge of the disease is a major challenge for prevention and control of HEV infection in Africa (*4*).

Gedaref State is in the southeastern region of Sudan, along the borders of Ethiopia and Eritrea (Appendix Figure). In early 2022, the area was hosting >60,000 refugees who fled from the Tigray War in Ethiopia. After arriving at the reception camp in Hamdayet, Sudan, the refugees were assigned to 1 of 3 long-term humanitarian camps: Tunaydbah, Um Rakuba, or Village 8 (*5*). During recent years, the region has had severe weather events, including heavy rains and flooding, that increased risks for infectious disease outbreaks (*5*,*6*).

On June 2, 2021, cases of acute jaundice syndrome appeared among the refugees in the Um Rakuba camp and were reported from the other humanitarian camps 2 weeks later. Patients were 3 months–64 years of age, and most (50.1%) were 16–30 years of age; 81 (5.2%) patients were <5 years of age, and 95 (6.1%) were >50 years of age. The male to female ratio was 1.9:1. Of 1,589 patients, 100% had jaundice; 83% had yellowish urine; and 78% had anorexia, nausea, and fatigue. Other symptoms included fever (61%), abdominal pain (56%), and headache and vomiting (44%). Among 22 initial acute jaundice syndrome cases, samples from 14 (64%) patients tested positive for HEV at the National Public Health Laboratory in Khartoum, Sudan, by using real-time PCR kits (Altona Diagnostics, https://www.altona-diagnostics.com). The outbreak appeared to peak in July 2021 during which 395 cases were reported (Figure). By February 21, 2022, ≈1,589 cases that included 21 pregnant women and 1 fatality (nonpregnant woman) were identified by using the Rapid Anti-HEV-IgM Test (InTec Products, https://www.intecasi.com) (Figure). Most (75%) cases were reported from the Um Rakuba camp (Appendix).

**Figure:**
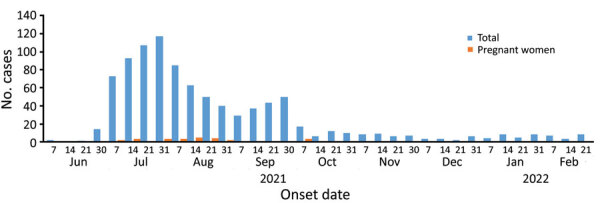
Number of cases of HEV infections per week among Tigray War refugees from Ethiopia in Sudan, June 2, 2021–February 21, 2022. HEV infections occurred in 3 humanitarian camps for refugees in Gedaref State, Sudan. The HEV outbreak peaked in July 2021 during which 395 cases were reported. HEV, hepatitis E virus.

The HEV outbreak in Sudan was associated with heavy rainstorms that flooded the humanitarian settlements and destroyed >1,231 latrines and >1,500 family shelters (*5*). A similar HEV outbreak occurred among refugees from South Sudan hosted in humanitarian camps in western Ethiopia, where >1,000 cases and a 2% mortality rate were reported (*7*). However, we report a relatively low mortality rate of <0.1% (1/1,589). Among pregnant women attending antenatal clinics in Tigray, Ethiopia, in 2018, lower hygiene and rural residency were associated with a high (43.4%) HEV seroprevalence, suggesting that a large outbreak could have been prevented by improving hygienic conditions (*4*). 

HEV vaccination is recommended for preventing and controlling HEV outbreaks in humanitarian settings, particularly for pregnant women (*1*,*3*). However, the success of vaccination is dependent on the HEV genotype. Because of limited resources, we were unable to genotype the HEV that was circulating in the camps. 

Recent outbreaks of Rift Valley fever in northern Sudan and dengue fever in western Sudan have occurred (*8*–*10*). These outbreaks highlight the association between massive population displacements because of war or armed conflict and the emergence of infectious diseases (*5*,*6*,*8*–*10*). Most (50%) HEV outbreaks in sub-Saharan Africa have occurred among refugees and displaced persons living in humanitarian crisis settings (*3*,*4*). Open defecation and flooding, both of which occur in the camps, are additional risk factors for HEV emergence and can lead to contamination of nearby open sources of drinking water and food (*5*). 

In summary, we report an outbreak of HEV infection among refugees from Ethiopia hosted in humanitarian camps in Gedaref State, Sudan. Implementing HEV vaccination, water, sanitation, and hygiene programs to improve the living conditions and drinking water among refugees and displaced persons in these camps might reduce the risk for HEV outbreaks. In addition, genotyping circulating HEV could clarify virus transmission routes and inform control measures. 

Appendix Additional information on hepatitis E virus outbreak among Tigray War refugees from Ethiopia, Sudan.
