# Supplementary material for: Hepatitis E Virus Outbreak among Tigray War Refugees from Ethiopia, Sudan
Source: Emerg Infect Dis. 2022 Aug;28(8):1722–4. doi: 10.3201/eid2808.220397 (PMC9328910; doi:10.3201/eid2808.220397)
Supplement: Appendix — Additional information on hepatitis E virus outbreak among Tigray War refugees from Ethiopia, Sudan. [file 22-0397-Techapp-s1.pdf]

Article DOI: https://doi.org/10.3201/eid2808.220397

# Hepatitis E Virus Outbreak among Tigray War Refugees from Ethiopia, Sudan

## Appendix

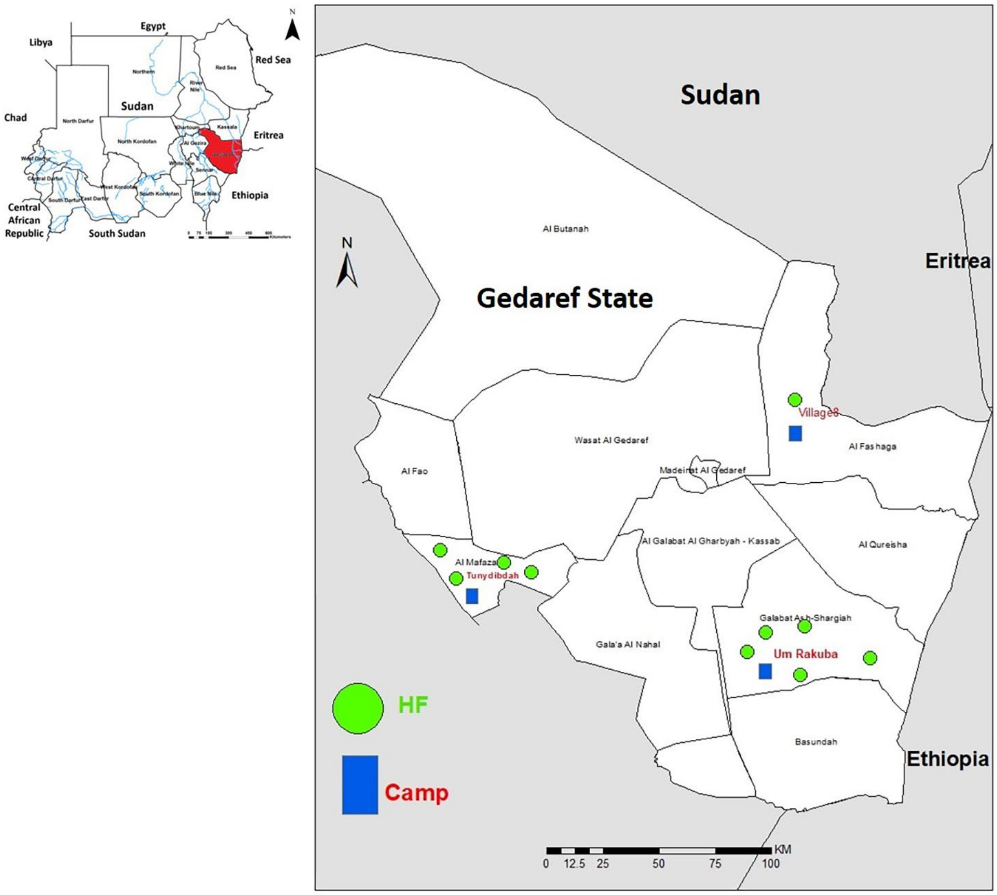


**Appendix Figure.** Location of hepatitis E virus outbreaks among Tigray War refugees from Ethiopia in Gedaref State, Sudan. Inset shows the location of Gedaref State (in red) in the southeastern region of Sudan along the borders of Ethiopia and Eritrea. Blue squares indicate 3 humanitarian camps that hosted Tigray War refugees from Ethiopia during June 2, 2021–February 21, 2022. Green circles indicate health facilities where cases of HEV infection were reported. HEV outbreaks were reported in all 3 camps. HEV, hepatitis E virus; HF health facilities.
